# Tricuspid regurgitation risk scores in patients undergoing tricuspid valve transcatheter edge‐to‐edge repair

**DOI:** 10.1002/ejhf.3599

**Published:** 2025-02-05

**Authors:** Cecilia Ennin, Lukas Stolz, Thomas J. Stocker, Ludwig T. Weckbach, Philipp M. Doldi, Julia Novotny, Jonas Gmeiner, Michael Näbauer, Steffen Massberg, Jörg Hausleiter

**Affiliations:** ^1^ Medizinische Klinik und Poliklinik I, Klinikum der Universität München Munich Germany; ^2^ German Center for Cardiovascular Research (DZHK), Partner Site Munich Heart Alliance Munich Germany

Tricuspid regurgitation (TR) is associated with increased morbidity, mortality and the risk of frequent heart failure hospitalizations. Tricuspid transcatheter edge‐to‐edge repair (T‐TEER) evolved as a powerful technique for the effective treatment of TR without the need for open‐heart surgery. Recent data of two randomized controlled trials demonstrated significant improvement of quality of life by T‐TEER in addition to optimal medical therapy.^1^, ^2^ Beyond that, safety and effectiveness of T‐TEER have been confirmed in several real‐world registries.^3^, ^4^, ^5^, ^6^ However, improvement of heart failure hospitalizations or mortality following T‐TEER has not been observed yet. Therefore, careful patient selection prior to T‐TEER is key to further optimize outcomes and potentially reduce mortality of heart failure patients with severe TR. Several TR risk scores are available which were mostly designed and validated to predict early survival prognosis in surgically or conventionally treated TR patients. The aim of this study was to evaluate the performance of six currently available risk scores in a large real‐world T‐TEER cohort for the prediction of 1‐year survival.

The study included T‐TEER patients treated at a high‐volume heart valve centre over a time period of 7 years (2016–2023) with available information necessary to calculate the risk scores of interest (TRI‐SCORE, Wang score, STS score, TRIO score, TriValve score, LaPar score).^7^, ^8^, ^9^, ^10^, ^11^, ^12^, ^13^ Thresholds in terms of high‐risk prediction were set at ≥10 points for the TRI‐SCORE and Wang score, at ≥8 points for the TRIO and LaPar scores and at ≥2.5 points for the TriValve score as suggested in the respective publications. Since no official high‐risk prediction threshold for the STS score exists, score values beyond the third quartile (>5.0%) were considered as cut‐off. T‐TEER was performed using either the PASCAL device (Edwards Lifesciences, Irvine, CA, USA) or the MitraClip/TriClip system (Abbott, Santa Clara, CA, USA). The primary study endpoint was 1‐year all‐cause mortality after T‐TEER. The analysis was performed in line with the principles outlined in the Declaration of Helsinki and received respective ethical oversight.

**Figure 1:**
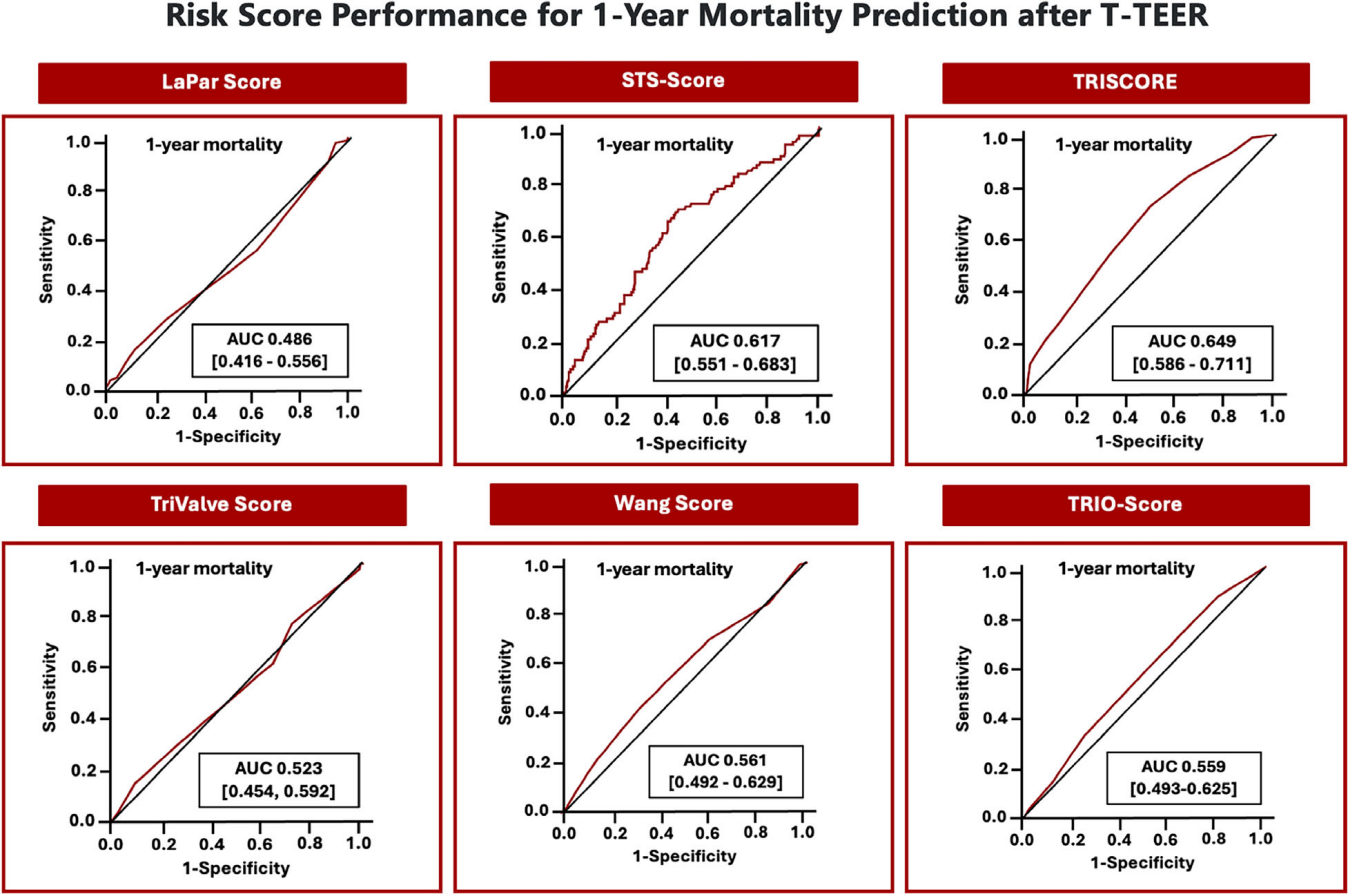
Risk score performance for 1‐year mortality prediction after tricuspid transcatheter edge‐to‐edge repair (T‐TEER). AUC, area under the curve.

The study included 385 patients at a mean age of 78.0 ± 8.6 years (49.6% female). Baseline TR was graded 5+ in 12.7%, 4+ in 32.2% and 3+ in 51.2% of patients. Mean left ventricular ejection fraction was overall preserved (53.4 ± 12.7%), tricuspid annular plane systolic excursion was 18.0 ± 5.1 mm. T‐TEER successfully reduced TR to grade ≤2+ in 87.0% of patients at discharge. Overall 1‐year survival was 76.4%. Patients presented with a median TRI‐SCORE of 6 (4–7) points, a median TRIO score of 6 (5–7) points, a median Wang score of 5 (4–6) points, a median TriValve score of 2 (1–2) points, a median LaPar score of 6 (5–7) points, and a median STS score of 3.2% (2.2–5.0%). The area under the curve (AUC) was determined to be 0.649 (0.586–0.711) for the TRI‐SCORE, 0.561 (0.492–0.629) for the Wang score, 0.559 (0.493–0.625) for the TRIO score, 0.523 (0.454–0.592) for the TriValve score, 0.486 (0.416–0.556) for the LaPar score, and 0.617 (0.551–0.683) for the STS score (*Figure* *1*).

Applying the above‐mentioned thresholds for identification of a sub‐population of patients in which a T‐TEER procedure might be high risk or even futile identified 103 patients (26.7%) according to the TriValve score, 1 patient (0.3%) according to the Wang score, 20 patients (5.1%) according to the TRI‐SCORE, 50 patients (13.0%) according to the TRIO score, 49 patients (12.8%) according to the LaPar score, and 97 patients (25.2%) according to the STS score as high‐risk patient population. However, more than 70% of patients in the high‐risk groups according to each score were alive after 1 year. Based on the same thresholds no discrimination of 30‐day mortality was achieved (low‐ vs. high‐risk group: TRI‐SCORE 0.0% vs. 5.3%; STS score 0.0% vs. 1.0%; TRIO score 0.3% vs. 0.0%; Wang score 0.3% vs. 0.0%; TriValve score 0.0% vs. 1.0%; LaPar score 0.3% vs. 0.0%).

In this study, we present a comprehensive comparison of available TR risk scores within a real‐world cohort of T‐TEER patients. Although providing predictive value in terms of 1‐year mortality, AUC and thus predictive accuracy were comparably low for all investigated TR risk scores. Furthermore, the existing scores did not allow the identification of a sub‐population of T‐TEER patients in which the procedure might be high risk or even futile due to excessive 1‐year mortality rates. This further emphasizes the huge heterogeneity of TR patients treated by T‐TEER in real‐world clinical pratice.^14^ Dedicated scores for TR patients undergoing T‐TEER derived from large datasets with independent derivation and validation cohorts are needed to improve risk prediction with a highly comorbid and heterogeneous population. As already shown for mitral TEER, machine learning might enhance the predictive performance of future T‐TEER scores. Doing so might improve outcome prediction which potentially helps physicians and patients in an informed decision‐making process on what to expect from an interventional TR treatment on an individual patient level.
